# Prophage Integrase Typing Is a Useful Indicator of Genomic Diversity in *Salmonella enterica*

**DOI:** 10.3389/fmicb.2017.01283

**Published:** 2017-07-10

**Authors:** Anna Colavecchio, Yasmin D’Souza, Elizabeth Tompkins, Julie Jeukens, Luca Freschi, Jean-Guillaume Emond-Rheault, Irena Kukavica-Ibrulj, Brian Boyle, Sadjia Bekal, Sandeep Tamber, Roger C. Levesque, Lawrence D. Goodridge

**Affiliations:** ^1^Food Safety and Quality Program, Department of Food Science and Agricultural Chemistry, McGill University, Sainte-Anne-de-BellevueQC, Canada; ^2^Institut de Biologie Intégrative et des Systèmes, Université Laval, Quebec CityQC, Canada; ^3^Pathogènes entériques et Bioterrorisme, Laboratoire de santé publique du Québec, Sainte-Anne-de-BellevueQC, Canada; ^4^Salmonella Research Laboratory, Bureau of Microbial Hazards, Health Canada, OttawaON, Canada

**Keywords:** *Salmonella enterica*, foodborne pathogen, genome diversity, prophage integrase gene analysis, signature genes

## Abstract

*Salmonella enterica* is a bacterial species that is a major cause of illness in humans and food-producing animals. *S. enterica* exhibits considerable inter-serovar diversity, as evidenced by the large number of host adapted serovars that have been identified. The development of methods to assess genome diversity in *S. enterica* will help to further define the limits of diversity in this foodborne pathogen. Thus, we evaluated a PCR assay, which targets prophage integrase genes, as a rapid method to investigate *S. enterica* genome diversity. To evaluate the PCR prophage integrase assay, 49 isolates of *S. enterica* were selected, including 19 clinical isolates from clonal serovars (Enteritidis and Heidelberg) that commonly cause human illness, and 30 isolates from food-associated *Salmonella* serovars that rarely cause human illness. The number of integrase genes identified by the PCR assay was compared to the number of integrase genes within intact prophages identified by whole genome sequencing and phage finding program PHASTER. The PCR assay identified a total of 147 prophage integrase genes within the 49 *S. enterica* genomes (79 integrase genes in the food-associated *Salmonella* isolates, 50 integrase genes in *S*. Enteritidis, and 18 integrase genes in *S*. Heidelberg). In comparison, whole genome sequencing and PHASTER identified a total of 75 prophage integrase genes within 102 intact prophages in the 49 *S. enterica* genomes (44 integrase genes in the food-associated *Salmonella* isolates, 21 integrase genes in *S*. Enteritidis, and 9 integrase genes in *S*. Heidelberg). Collectively, both the PCR assay and PHASTER identified the presence of a large diversity of prophage integrase genes in the food-associated isolates compared to the clinical isolates, thus indicating a high degree of diversity in the food-associated isolates, and confirming the clonal nature of *S*. Enteritidis and *S*. Heidelberg. Moreover, PHASTER revealed a diversity of 29 different types of prophages and 23 different integrase genes within the food-associated isolates, but only identified four different phages and integrase genes within clonal isolates of *S.* Enteritidis and *S.* Heidelberg. These results demonstrate the potential usefulness of PCR based detection of prophage integrase genes as a rapid indicator of genome diversity in *S. enterica*.

## Introduction

*Salmonella enterica* is a Gram-negative pathogen that infects humans and animals. *S. enterica* is divided into six sub-species on the basis of genetic content (Wain and O’Grady, 2017), and contains more than 2,500 serovars. *S. enterica* subspecies *enterica* is a major cause of enteric disease in humans and animals. The majority of illnesses caused by *S. enterica* are foodborne. Globally, *S. enterica* causes 93 million gastroenteritis cases and 150,000 deaths annually (Majowicz et al., 2010). In Canada, salmonellosis accounts for 87,510 human cases, and 17 deaths each year (Thomas et al., 2013, 2015). However, while there are more than 2,500 serovars of *S. enterica* subspecies I, the majority (75%) of all salmonellosis cases in Canada are caused by only 10 serovars. And three serovars, *S.* Enteritidis (30%), *S*. Heidelberg (15%), and *S*. Typhimurium (12%), account for 57% of all salmonellosis cases in Canada (Public Health Agency of Canada [PHAC], 2012). In the United States, where *Salmonella* accounts for 1.2 million illnesses and 450 deaths annually (Scallan et al., 2011), the top 10 serovars cause approximately 57% of illnesses. Characterizing *Salmonella* serovars into monophyletic and polyphyletic lineages is essential for linking outbreaks (Timme et al., 2013). While the implementation of Hazard Analysis and Critical Control Point (HACCP) programs in the food industry has reduced contamination of foods of animal origin, there has been increased recognition of *Salmonella* contamination associated with fresh produce, which accounts for approximately half of all fresh produce outbreaks due to bacteria in the United States and European Union (Callejón et al., 2015).

These statistics have led to much work aimed at identifying virulence and fitness markers in *S. enterica*, as well as questions regarding genome diversity. *S. enterica* genomes are highly diversified due to insertions and deletions (indels) (Zhou et al., 2013). Survival in different habitats, as evidenced by the large number of host species colonized by *S. enterica*, and the ability to successfully transmit through food and water, or directly from host to host has driven this diversity. High levels of intra-serovar diversity has also been recognized, as demonstrated by the acquisition of indels (51 prophages, 10 plasmids, and 6 integrative conjugational elements) by *S. enterica* Agona (Zhou et al., 2013).

Prophages are bacteriophages which have integrated into bacterial chromosomes, by means of an integrase gene, and they have been found to contribute to interstrain genetic variability (Brüssow et al., 2004). Bacteriophages are the most abundant organisms on earth, and it is estimated that there are 10^31^ phage particles in the biosphere (McNair et al., 2012). Phages are ubiquitous and can be found in any environment where their bacterial hosts are present. It has been estimated that there are 100 million phage species (Rohwer, 2003). As such, phages likely play a major role in defining the dynamics of microbial community structure and function. In fact, much of the diversity observed in closely related bacterial strains is a result of the incorporation of diverse prophages into the core bacterial genome (Brüssow et al., 2004). Prophages enhance bacterial fitness by encoding many proteins important in virulence and antibiotic resistance.

Many studies have demonstrated the presence of numerous prophages within *S. enterica* (Figueroa-Bossi and Bossi, 1999; Kropinski et al., 2007). In one such study, Thomson et al. (2004) characterized prophages within the *S. enterica* serovar Typhi CT18 chromosome. *In silico* analyses were used to compare prophage regions in *S*. Typhi CT18, to prophages within 40 other *Salmonella* isolates using DNA microarray technology. The results indicated that the *S*. Typhi CT18 prophages had similarity to the lambda, Mu, P2 and P4 phage families. Other *S*. Typhi isolates also had similar prophages, supporting a clonal origin of this serovar. In contrast, distinct prophage variation was detected within a broad range of *Salmonella* serovars, suggesting that these phages may confer a level of specialization on their host. The authors concluded that prophages therefore play a crucial role in the generation of genetic diversity within *S. enterica*. This statement, and the lack of an universal phylogenetic marker for phages provides the rationale for this study in which we evaluated the use of prophage integrase genes as reliable indicators of genome diversity in *S. enterica*.

We compared two serovars (Enteritidis and Heidelberg) whose genomes are reported to be clonal, to a set of *S. enterica* isolates from diverse food sources (**Table 1**). These food associated isolates are considered to be “rare” because they belong to serovars that are not within the top 70 serovars that cause illness in Canada. We hypothesized that the integrase gene is associated with prophage diversity within *Salmonella*. Hence, screening integrase genes (via a PCR assay) would detect few and similar patterns of integrase genes in the non-diverse and clonal isolates of *S.* Enteritidis and *S.* Heidelberg but a larger number and diversity of integrase genes in the food associated isolates that are rare and diverse.

**Table 1:** List of *Salmonella enterica* isolates used in this study.

Taxon	Serovar	Salfos ID	Origin	Source isolation
*Salmonella enterica*	Amager	S25	HC^a^	Animal Feed
*Salmonella enterica*	Ball	S26	HC	Shellfish – Shrimp
*Salmonella enterica*	Banana	S27	HC	Animal Feed
*Salmonella enterica*	Bergen	S28	HC	Shellfish – Shrimp
*Salmonella enterica*	Broughton	S29	HC	Poultry
*Salmonella enterica*	Canada	S30	HC	Chocolate
*Salmonella enterica*	Casablanca	S31	HC	Fish/Shellfish
*Salmonella enterica*	Chingola	S32	HC	Fish – Seaweed
*Salmonella enterica*	Cremieu	S33	HC	Fish – Frozen eel
*Salmonella enterica*	Daytona	S34	HC	Shellfish – Clams
*Salmonella enterica*	Duesseldorf	S35	HC	Poultry
*Salmonella enterica*	Elisabethville	S36	HC	Reptiles – Agamid
*Salmonella enterica*	Falkensee	S37	HC	Spices – Rice seasoning
*Salmonella enterica*	Fresno	S38	HC	Animal feed
*Salmonella enterica*	Godesberg	S39	HC	Sesame – Halawa
*Salmonella enterica*	Hull	S40	HC	Fish – Dried conch
*Salmonella enterica*	Indikan	S41	HC	Sesame – Tahini
*Salmonella enterica*	Kouka	S42	HC	Shellfish – Oysters
*Salmonella enterica*	Luciana	S43	HC	Fruit – Cantaloupe
*Salmonella enterica*	Luckenwalde	S44	HC	Cocoa beans
*Salmonella enterica*	Orientalis	S45	HC	Alfalfa seeds
*Salmonella enterica*	Pasing	S46	HC	Chocolate
*Salmonella enterica*	Solt	S47	HC	Cocoa beans
*Salmonella enterica*	Tado	S48	HC	Animal feed
*Salmonella enterica*	Taiping	S49	HC	Fish
*Salmonella enterica*	Taksony	S50	HC	Ox
*Salmonella enterica*	Tyresoe	S51	HC	Shellfish – Shrimp
*Salmonella enterica*	Wentworth	S52	HC	Fish – Cuttlefish
*Salmonella enterica*	Westhampton	S53	HC	Animal feed supplement
*Salmonella enterica*	Weston	S54	HC	Shellfish – Shrimp
*Salmonella enterica*	Enteritidis	S3	LSPQ^b^	Clinical
*Salmonella enterica*	Enteritidis	S4	LSPQ	Clinical
*Salmonella enterica*	Enteritidis	S5	LSPQ	Clinical
*Salmonella enterica*	Enteritidis	S6	LSPQ	Clinical
*Salmonella enterica*	Enteritidis	S7	LSPQ	Clinical
*Salmonella enterica*	Enteritidis	S8	LSPQ	Clinical
*Salmonella enterica*	Enteritidis	S9	LSPQ	Clinical
*Salmonella enterica*	Enteritidis	S10	LSPQ	Clinical
*Salmonella enterica*	Enteritidis	S11	LSPQ	Clinical
*Salmonella enterica*	Enteritidis	S12	LSPQ	Clinical
*Salmonella enterica*	Heidelberg	S430	LSPQ	Clinical
*Salmonella enterica*	Heidelberg	S429	LSPQ	Clinical
*Salmonella enterica*	Heidelberg	S371	LSPQ	Clinical
*Salmonella enterica*	Heidelberg	S426	LSPQ	Clinical
*Salmonella enterica*	Heidelberg	S427	LSPQ	Clinical
*Salmonella enterica*	Heidelberg	S370	LSPQ	Clinical
*Salmonella enterica*	Heidelberg	S431	LSPQ	Clinical
*Salmonella enterica*	Heidelberg	S432	LSPQ	Clinical
*Salmonella enterica*	Heidelberg	S433	LSPQ	Clinical


*Further details can be found in the SalFoS database at https://salfos.ibis.ulaval.ca/.^a^Health Canada, Ottawa, ON, Canada. ^b^Laboratoire de santé publique du Québec, Sainte-Anne-de-Bellevue, QC, Canada*.

## Materials and Methods

### Bacterial Isolates and Growth Conditions

Forty-nine *S. enterica* isolates (**Table 1**) were used in this study. Metadata for all *S*. enterica isolates can be found in the *Salmonella* Foodborne Syst-OMICS Database (SalFoS), which can be accessed at https://salfos.ibis.ulaval.ca/. In order to determine whether prophage integrase genes could be used to assess genome diversity in *S. enterica*, we chose isolates from serovars that are consistently implicated in outbreaks of salmonellosis and that are also clonal in nature (clinical isolates from serovars Enteritidis and Heidelberg) as well as isolates from rare serovars that were isolated from diverse food sources (food associated isolates). All isolates were maintained at -80°C in glycerol, and were revived by streaking the frozen culture on tryptic soy agar (TSA) (BD Biosciences, Mississauga, ON, Canada) followed by incubation at 37°C for 16 h. Isolated colonies were then inoculated in tryptic soy broth (TSB) (BD Biosciences, Mississauga, ON, Canada) and grown at 37°C for 16 h in an orbital shaker at a speed of 225 rpm.

### DNA Extraction and Amplification of Bacteriophage Specific Integrase Genes

Bacterial DNA was extracted from an overnight TSB culture using the DNeasy Blood and Tissue kit (Qiagen Inc., Germantown, MD, United States) according to the manufacturer’s instructions. The polymerase chain reaction (PCR) was used to amplify prophage tyrosine integrase genes using a prophage integrase assay previously described by Balding et al. (2005). Briefly, a set of 11 degenerate primer sets were designed by aligning the conserved regions, designated as “box I” and “box II,” of the tyrosine integrase of 32 enteric prophages encoded by members of the *Enterobacteriaceae* family. The two conserved regions are located in the C-terminal of the tyrosine integrase and consist of residues A202-G227 (“Box I”) and T206-D344 (“Box II”) in the Lambda prophage. Prophages encoding an integrase gene with similar “box I” and “box II” regions were grouped into eight primer sets. Primer set 5 was further subdivided into groups 5A, 5B, and 5C, and group 6 was further subdivided into groups 6A and 6B, for a total of 11 degenerate primer sets. The primer sets and sequences can be found in Balding et al. (2005).

Polymerase chain reaction amplification was conducted in a Peltier Thermal Cycler (PTC-100, Bio-Rad Laboratories, Inc., Mississauga, ON, Canada), and commenced with DNA denaturation for 5 min at 94°C followed by 25 cycles consisting of 94°C for 30 s, 40°C for 30 s, 72°C for 30 s and a final extension at 72°C, for 7 min. PCR amplicons ranged in size from 280 to 447 bp, and were resolved by electrophoresis in 1X Tris/Borate/EDTA (TBE) buffer on 1% (w/v) agarose gels that contained 1x SYBR Safe stain (Thermo Fisher Scientific, Waltham, MA, United States). Following gel electrophoresis, amplicons were visualized under UV illumination.

### Whole Genome Sequencing and Bioinformatic Analysis

Whole genome sequencing was performed at the EcoGenomics Analysis Platform (IBIS, Université Laval, Quebec City, QC, Canada) on an Illumina MiSeq using 300-bp paired-end libraries with 40× coverage. The raw reads were assembled using the A5 pipeline (Tritt et al., 2012). Each of the 49 assembled genomes were analyzed by PHASTER to identify the presence of prophages and their integrase genes (Arndt et al., 2016). Only prophages identified as “complete” or “intact” were considered for further analysis. The identity of all intact prophage sequences detected by PHASTER was confirmed by BLAST (Altschul et al., 1990).

### Phylogenetic Tree Construction

Parsnp, included in the Harvest suite of core-genome alignment tools, was performed to produce a rapid core-genome alignment based on SNPs (1000 bootstraps) of the core genome sequences of the 30 food associated *Salmonella* isolates and 19 clinical *S.* Enteritidis and *S*. Heidelberg isolates (Treangen et al., 2014). The alignment data was converted to Newick format and a unrooted maximum-likelihood tree was constructed and edited with Interactive tree of life (iTOL) version 3 (Letunic and Bork, 2016). Whole genome alignments of the prophage sequences as well as their integrase genes, and construction of unrooted maximum-likelihood trees were performed using BioNumerics version 7.6.2 (Applied Maths, 2017). All phylogenetic trees constructed using BioNumerics were converted to Newick files and edited with iTOL (Letunic and Bork, 2016).

## Results

### Whole Genome Alignment Reveals Clonality of Clinical *Salmonella* Enteritidis and *Salmonella* Heidelberg Isolates

**FIGURE 1:**
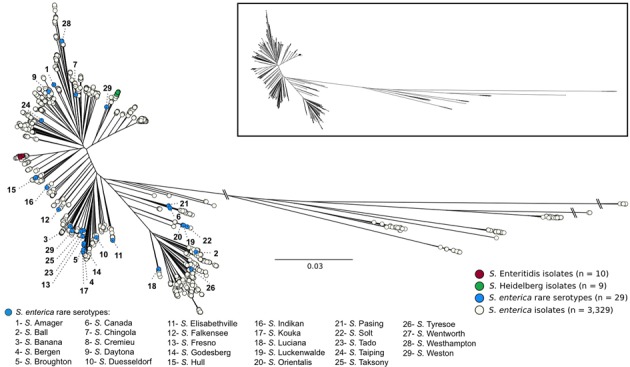
Unrooted maximum likelihood tree of 3,377 *Salmonella enterica* genomes based on 196,774 SNPs from 839 core genes and using FastTree 2.1.9 (1,000 bootstraps). The dendrogram has been modified from a previous publication (Emond-Rheault et al., 2017).

Whole genome sequences (WGS) of all *S. enterica* isolates evaluated in this study are contained within the SalFoS database. To investigate the potential for the PCR assay to be used as a rapid screening tool to determine genome diversity in *Salmonella*, we compared the results of the PCR assay with core genome SNP sequence alignments for a population of clonal and diverse S. enterica isolates chosen from the SalFoS database. Previously, we sequenced the whole genomes of 3,337 *S. enterica* isolates contained within the SalFoS database, and aligned their core genomes based on SNPs (Emond-Rheault et al., 2017). This data was used to construct an unrooted maximum-likelihood tree of the core genome sequences. Core genome alignment is a subset of whole-genome alignment, which facilitates the construction of large phylogenetic trees between related microorganisms by using the essential genes contained within the core genome (Treangen et al., 2014). Single-nucleotide polymorphisms (SNPs) within the core genome are the most reliable variant to infer large phylogenetic relationships between closely related microorganisms (Treangen et al., 2014). We used core-genome SNP analysis to study the evolution and diversity of *Salmonella*, and observed that *S. enterica* isolates were grouped within two clades, while *Salmonella* from other subspecies clustered within separate clades (Emond-Rheault et al., 2017). These results are in agreement with those of Timme et al. (2013), who studied 156 WGS of *Salmonella* from the six *S. enterica* subspecies by core SNP analysis and observed two clades of *S. enterica* subspecies *enterica*. From our phylogenetic tree, we selected 30 genomically diverse, food associated *Salmonella* isolates from rare serovars (one isolate per serovar), and a clonal population of *Salmonella* consisting of 10 clinical *S.* Enteritidis isolates and 9 clinical *S.* Heidelberg isolates. The food associated *Salmonella* serovars were dispersed throughout the tree (**Figure 1**) suggesting that these isolates are genetically diverse and could be easily distinguished at the serovar level. In contrast, the *S.* Enteritidis and *S.* Heidelberg isolates were clustered within their corresponding serovar branch with low genetic diversity among strains underscoring the clonal nature of these serovars (Deng et al., 2015; Labbé et al., 2016).

### Bioinformatics Analysis Reveals Diversity of Prophages in Rare *Salmonella* Isolates

**FIGURE 2:**
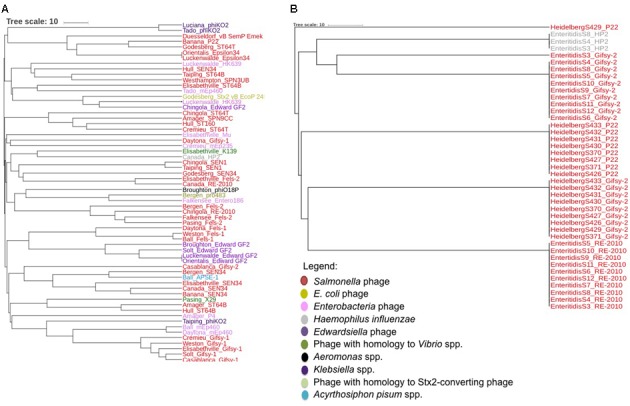
**(A)** Unrooted maximum-likelihood tree of 61 prophages encoded by isolates of rare *Salmonella* serovars identified by PHASTER and based on a multiple alignment as defined by BioNumerics version 7.6.2. **(B)** Unrooted maximum-likelihood tree of 41 prophages encoded by *Salmonella* Enteritidis and *Salmonella* Heidelberg identified by PHASTER based on a multiple alignment as defined by BioNumerics version 7.6.2.

The prophage finding software PHASTER was used to identify intact prophages and their integrase genes within the food associated and clinical *Salmonella* isolates. A total of 102 intact prophages and 75 integrase genes (PHASTER was unable to identify an integrase gene in some prophages) were identified by PHASTER. Additionally, PHASTER identified the presence of a large diversity of prophages in the food-associated isolates compared to the clinical isolates (**Figure 2**). For example, PHASTER identified 29 different types of prophages (and 23 different integrase genes) from nine different bacterial species among *S. enterica* food associated isolates (**Figure 2A**). The majority of the prophages infect *S. enterica*, while other prophages originate from *Escherichia coli, Enterobacteria, Haemophilus influenzae*, *Edwardsiella* spp., *Aeromonas* spp., *Klebsiella* spp., and *Acyrthosiphon spium*. In addition, PHASTER identified two prophages with high homology to *Vibrio* spp. in a *Salmonella* Pasing isolate and a *Salmonella* Elisabethville isolate, and one prophage with high homology to a shiga toxin 2 (Stx2) converting phage in an isolate from serovar Godesberg. In contrast, PHASTER identified only four different types of prophages and four integrase genes within *S.* Enteritidis and *S.* Heidelberg (**Figure 2B**). The four prophages are the common *Salmonella* phages P22, Gifsy-2, and RE-2010, and the *Haemophilus influenzae* phage HP2.

### The PCR Assay Reveals Genetic Diversity of Prophage Integrase Genes in Food Associated *Salmonella* Isolates

**FIGURE 3:**
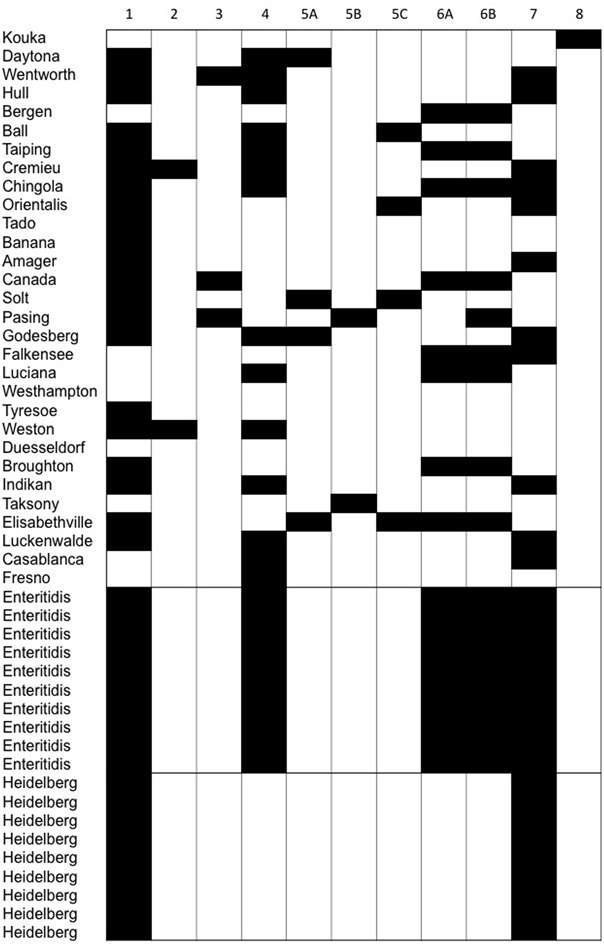
The diversity of phage integrase genes detected by PCR within isolates of rare *Salmonella* serovars compared to the minimal diversity of phages identified within clonal *Salmonella* serovars Enteritidis and Heidelberg.

The PCR assay targets the tyrosine integrase genes of 32 enteric phages infecting members of the *Enterobacteriaceae* family. Using the PCR assay, a total of 147 integrase genes (79 integrase genes in the food associated *Salmonella* isolates, 50 integrase genes in *S*. Enteritidis, and 18 integrase genes in *S*. Heidelberg) were identified. In agreement with the core genome sequence analysis, the integrase genes from the food associated *Salmonella* isolates were much more diverse than the integrase genes in the clinical *Salmonella* isolates. For example, all 11 primer sets produced amplicons in the food associated isolates, while only five primer sets (1, 4, 6A, 6B, and 7) produced amplicons in the *S*. Enteritidis isolates, and only two primer sets (1 and 7) produced amplicons in the *S*. Heidelberg isolates. Furthermore, all *S*. Enteritidis isolates contained the same prophage integrases, a result that was also observed in the *S*. Heidelberg isolates. Prophage integrase genes amplified by primer sets 2, 3, 5A, 5B, 5C, and 8 were detected in the food associated *Salmonella* isolates, but not the clinical (*S*. Enteritidis and *S*. Heidelberg) isolates (**Figure 3**).

### The Integrase Gene Is an Indicator of Prophage Diversity in *Salmonella*

**FIGURE 4:**
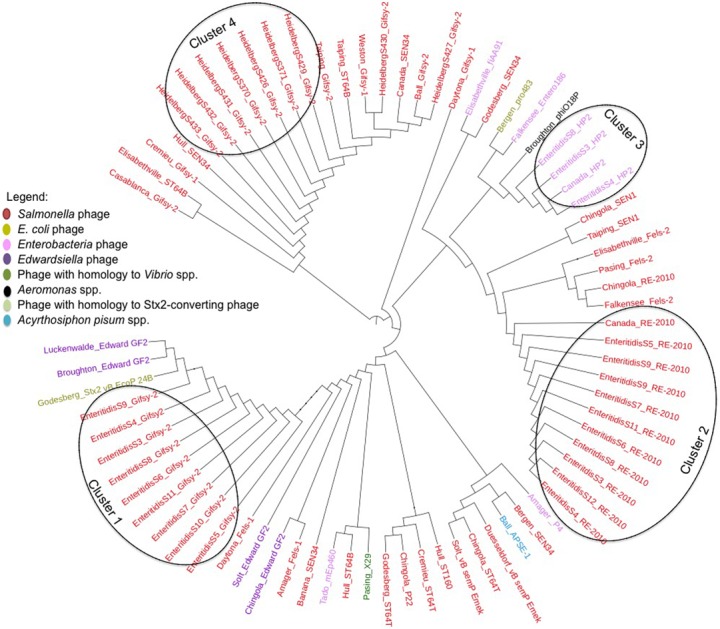
Unrooted maximum-likelihood tree of 75 tyrosine integrase genes of prophages encoded within all 49 *Salmonella enterica* isolates identified by PHASTER and based on a multiple alignment as defined by BioNumerics version 7.6.2.

To further demonstrate that the PCR assay can reveal prophage diversity in *Salmonella* via the integrase gene, a multiple alignment of the integrase gene of the intact prophages identified by PHASTER was performed and used to construct a maximum-likelihood tree (**Figure 4**). Nine of ten *S*. Enteritidis isolates contained intact Gifsy-2 prophages, and the integrase genes from these phages all clustered together (**Figure 4**, Cluster 1). Additionally, all *S*. Enteritidis isolates also contained intact RE-2010 prophages, and the integrase genes of these phages clustered together (**Figure 4**, Cluster 2) in a similar fashion to that observed with the Gifsy-2 integrase genes. These results suggest that the integrase genes from Gifsy-2 and RE-2010 phages are clonal within the isolates of this serovar, and demonstrate that the use of the integrase gene is in agreement with the use of core genome SNP analysis of *S*. Enteritidis, as predictors of genome diversity. The results also indicate that bioinformatic analysis of all prophage integrase genes contained within a given isolate may be used to add discrimination to core genome SNP analysis studies, in cases where the genomes are clonal in nature, as is the case with *S*. Enteritidis. For example, three *S*. Enteritidis isolates (S3, S4, and S8) contain an integrase from the HP2 prophage (**Figure 4**, Cluster 3), which is not found in the other *S*. Enteritidis isolates, and therefore could be used to differentiate between the isolates.

Similar results were observed with the *S*. Heidelberg isolates. For example, all *S*. Heidelberg isolates also contain Gifsy-2 prophages, but the sequence of their integrase genes are different from those contained within the *S*. Enteritidis isolates (**Figure 4**, Cluster 4). Also, while the majority (seven of nine Gifsy-2 integrase genes) formed a cluster, the Gifsy-2 integrase genes from two isolates, *S*. Heidelberg S427 and S430 are outliers and cluster with other similar prophage integrase genes (**Figure 4**). Taken collectively with the *S*. Enteritidis data, these results demonstrate that single integrase genes within a respective isolate can be used to assess genomic diversity, while the collective number of integrase gene sequences contained within an isolate can be used for discrimination among genetically similar isolates.

## Discussion

The study of phage diversity trails far behind similar studies of the bacterial and eukaryotic kingdoms, and only a small fraction of phages have so far been characterized (Adriaenssens and Cowan, 2014). This is largely due to the absence of any universal phylogenetic marker (or signature gene) for phages. Unlike bacteria, in which the 16S rRNA gene is a universal gene that can be used for taxonomy and phylogeny, phages have no universally present gene that can be used for taxonomic analysis (Rohwer and Edwards, 2002). Various signature genes have been used to investigate phage diversity, including genes encoding structural proteins (portal proteins, major capsid proteins, and tail sheath proteins), auxiliary metabolism genes (*psbA*, *psbB*, and *phoH*), and several polymerase genes (Adriaenssens and Cowan, 2014). The majority of work conducted on signature gene analysis to assess phage diversity has focused on virulent phages. With respect to diversity conferred on bacterial hosts by temperate phages, Verghese et al. (2011) investigated the use of comK prophage junction fragments as markers for *Listeria monocytogenes* genotypes that persisted in individual meat and poultry processing plants (Verghese et al., 2011). In this work, the authors demonstrated that sequences in comK prophage junction fragments could be used to differentiate strains of epidemic clones (ECs), which, when identified, were shown to be specific to individual meat and poultry processing plants. The authors concluded that comK prophage junction fragment sequences may permit accurate tracking of persistent strains within individual food processing operations and thus allow the design of more effective intervention strategies to reduce contamination and enhance food safety (Verghese et al., 2011).

The absence of a universal marker for phages and the fact that that signature genes are only specific for certain phages or phage species, limits the use of such approaches to related phages, meaning that these approaches are not useful when assessing phages that are genetically diverse. Hence, in this work, we used the prophage integrase gene as a signature gene to measure prophage diversity, and therefore the diversity of host genomes that carry the prophages. Many factors such as pathogenicity islands, virulence factors, and antimicrobial and heavy metal resistance genes are encoded by prophages and provide genetic diversity among bacterial species (Brüssow et al., 2004). We therefore hypothesized that prophages may contribute to the diversity in the *Salmonella* isolates from rare serovars, and a lack of diversity in the clonal (clinical) serovars. The tyrosine integrase gene can be used as an indicator for phage diversity because it is carried by over 300 prophages (Balding et al., 2005). Although prophages can carry serine integrase genes, only approximately 30 members have been identified and they are not typically carried by foodborne bacteria (Fogg et al., 2014). The C-terminal of the tyrosine integrase contains two highly conserved regions designated “box 1” and “box II.” The tyrosine residue is located at residue 342 in “box II” and is conserved in every family member encoding a tyrosine integrase and is responsible for the DNA cleavage on the bacterial attachment site (attB) and the phage attachment site (attP), which facilitate the integration of the prophage into its host (Balding et al., 2005). In addition, two arginine residues R212 and R311 that assist in the facilitation of prophage integration are also conserved in all family members. Bobay et al. (2013) investigated the number and type of integration loci of prophages within *E. coli* and *S. enterica* genomes. They observed that the most prevalent (46%) integration site flanking prophages in *S. enterica* was a protein coding sequence, *lepA*, followed by tRNA and tmRNA genes (37%) and sRNA genes (17%). Of 24 prophage integration sites observed within *S. enterica*, 19 were also present in *E. coli*, suggesting that prophage integration sites are restricted to a few bacterial sites. The authors also observed that 423 of 500 prophages (83%) contained an integrase, of which all were tyrosine integrases, and a phylogenetic analysis demonstrated that closely related integrases integrate at the same integration sites. Since phages restrict their integration to conserved sites on the host genome, this further supports our findings that the integrase gene can serve as an indicator of prophage diversity (Bobay et al., 2013).

Core genome SNP analysis of the *Salmonella* isolates demonstrated a high degree of clonality in the *S*. Enteritidis and *S*. Heidelberg isolates (**Figure 1**), in agreement with previous studies, while also showing that the food associated *Salmonella* were highly diverse. Ogunremi et al. (2014) observed the clonality of 11 *S.* Enteritidis isolates, and demonstrated that they were all characterized by a comparably sized genome, an estimated 23-905 SNPs among genome pairs and five prophages or prophage remnants. Hoffmann et al. (2014) observed the clonality of 44 *S.* Heidelberg isolates, in which nearly 30 were indistinguishable by pulsed-field gel electrophoresis (PFGE), had significantly fewer core genome SNPs then *S.* Newport and, *S.* Typhimurium and contained similar prophages.

In this study, the results of the core genome SNP analysis agreed with a prophage integrase gene bioinformatic approach, in which prophages and integrase genes were identified within WGS using PHASTER, and used to construct unrooted maximum likelihood trees. The integrase gene was a good predictor of the diversity of the entire prophage sequence, as prophages that clustered together (i.e., Gifsy-2, HP2, and RE-2010) (**Figure 2**), contained integrase genes that also clustered together (**Figure 4**). Gifsy-2 is a highly studied phage known to integrate into various strains of *S.* Typhimurium. HP2 is a P2-like phage known to infect *Salmonella* spp., while RE-2010 has high nucleotide homology to Fels-2 prophages that have been observed in various *Salmonella* genomes (Switt et al., 2015). Also, both the entire prophage sequences and the integrase gene sequences were good predictors of *Salmonella* core genome diversity, as only four intact prophages and their integrase genes were identified in the clonal *Salmonella* (Enteritidis and Heidelberg) serovars, while 29 different types of prophages and 23 different integrase genes, were identified in the food associated *Salmonella* isolates. These results agreed with the core genome SNP analysis of the *Salmonella* isolates, in which similar results regarding the genome diversity of the clinical and food associated isolates were observed.

**FIGURE 5:**
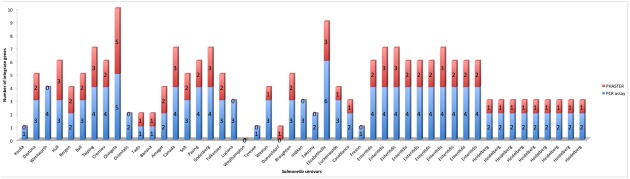
Comparison of the number of integrase genes detected by the PCR assay to the number of integrase genes detected by PHASTER within all 49 *Salmonella enterica* genomes.

As with the bioinformatics approach, the PCR assay detected more integrase genes in the food associated isolates than the clinical isolates. Additionally, when the PCR assay was used to evaluate the *Salmonella* isolates for the presence of integrase genes, the results showed that the PCR assay detected 147 integrases in the 49 *Salmonella* isolates, while 74 integrases were detected by PHASTER in the intact prophages encoded by the *Salmonella* isolates (**Figure 5**). The differential ability of the two methods to detect prophage integrase genes is due to the fact that the primer sets used in the PCR assay detected the integrase genes of intact and cryptic phages, while our bioinformatic analysis focused only on the intact phages detected by PHASTER. The prophages encoded by the *Salmonella* isolates were identified as intact, questionable or incomplete by PHASTER. Incomplete phages suggest that they may represent cryptic phages, which may no longer encode an integrase gene. Questionable phages do not contain sufficient prophage genes to be considered complete functional phages. Thus, only intact phages were selected and blasted for confirmation to ensure an accurate multiple sequence alignment.

Other groups have demonstrated the use of prophages as markers of diversity in their bacterial hosts. For example, (Shan et al., 2012) conducted phylogenetic analysis of the holin sequences of *Clostridium difficile* prophages, and identified three groups of *C. difficile* phages, two within the Myoviridae and a divergent group within the Siphoviridae. The marker also produced homogenous groups within temperate phages that infect other taxa, including *Clostridium perfringens*, *Clostridium botulinum*, and *Bacillus* spp., indicating the potential use of the holin gene to study prophage carriage in other bacteria. This study demonstrated the high incidence of prophage carriage and diversity in clinically relevant strains of *C. difficile*.

Basu et al. (2000) conducted a molecular analysis of CTX prophages in clinical, classical biotype strains of *Vibrio cholerae* O1 that were isolated between 1970 and 1979 (Basu et al., 2000). Restriction fragment length polymorphism (RFLP) of rRNA genes and PFGE showed clonal diversity among the strains. The authors observed that one strain (GP13) had three CTX prophages while another (GP147) had four CTX prophages, indicating heterogeneity in the arrangement of the CTX prophages among classical strains of *V. cholerae* O1.

Collectively, our data shows that the prophage integrase PCR assay may be a good indicator of genome diversity in *S. enterica*, and that the PCR assay is a rapid and cost-effective rapid screening tool that may be used as a high throughput screen to evaluate large numbers of *Salmonella* isolates as a way to reduce the numbers of isolates that are submitted for whole genome sequencing to evaluate genomic diversity.

## Author Contributions

SB and ST supplied bacterial isolates and AC, YD, ET, LG, J-GE-R, JJ, LF, IK-I, and RL performed the analyses and drafted the manuscript. BB provided support for sequencing and analysis. All authors revised the manuscript.

## Conflict of Interest Statement

The authors declare that the research was conducted in the absence of any commercial or financial relationships that could be construed as a potential conflict of interest. The handling Editor declared a shared affiliation, though no other collaboration, with one of the authors ST, and the handling Editor states that the process met the standards of a fair and objective review.
